# Portable Analytical Techniques for Monitoring Volatile Organic Chemicals in Biomanufacturing Processes: Recent Advances and Limitations

**DOI:** 10.3389/fchem.2020.00837

**Published:** 2020-09-11

**Authors:** Xiaofeng Chen, Runmen Hu, Luoyu Hu, Yingcan Huang, Wenyang Shi, Qingshan Wei, Zheng Li

**Affiliations:** ^1^Institute for Advanced Study, Shenzhen University, Shenzhen, China; ^2^Department of Chemical and Biomolecular Engineering, North Carolina State University, Raleigh, NC, United States

**Keywords:** biomanufacturing, volatile organic chemicals, bioprocess assessments, analytical methods, electronic noses, sensors

## Abstract

It is essential to develop effective analytical techniques for accurate and continuous monitoring of various biomanufacturing processes, such as the production of monoclonal antibodies and vaccines, through sensitive and quantitative detection of characteristic aqueous or gaseous metabolites and other analytes in the cell culture media. A comprehensive summary toward the use of mainstream techniques for bioprocess monitoring is critically reviewed here, which illustrates the instrumental and procedural advances and limitations of several major analytical tools in biomanufacturing applications. Despite those drawbacks present in modern detection systems such as mass spectrometry, gas chromatography or chemical/biological sensors, a considerable number of useful solutions and inspirations such as electronic or optoelectronic noses can be offered to greatly overcome the restrictions and facilitate the development of advanced analytical techniques that can target a more diverse range of key nutritious components, products or potential contaminants in different biomanufacturing processes.

## Introduction

The industry of cell culture manufacturing has grown rapidly over the past decades. With the continuous emergence of state-of-the-art bioprocess technologies, numerous types of biological products, including therapeutic proteins, clinical enzymes, viral or recombinant vaccines, gene therapy vectors, and cells, have been successfully manufactured and put into real-world applications (Carrondo et al., 2012; Pais et al., 2014; Dumont et al., 2016; Lalonde and Durocher, 2017). In principle, conditions required for cell culture cultivation can be optimized and precisely controlled for large-scale industrial production. Nonetheless, cellular metabolism is a complicated process of physical and chemical changes that is subject to various environmental factors (e.g., temperature, O_2_, pH) (Nielsen and Keasling, 2016). Unexpected disruption in cell behavior due to unknown contaminations could strongly impair quality and reproducibility of bioproducts. Therefore, uncertainty and unpredictability in biopharmaceutical manufacture has posed a great challenge in field monitoring of relevant biological reactions.

**Figure 1:**
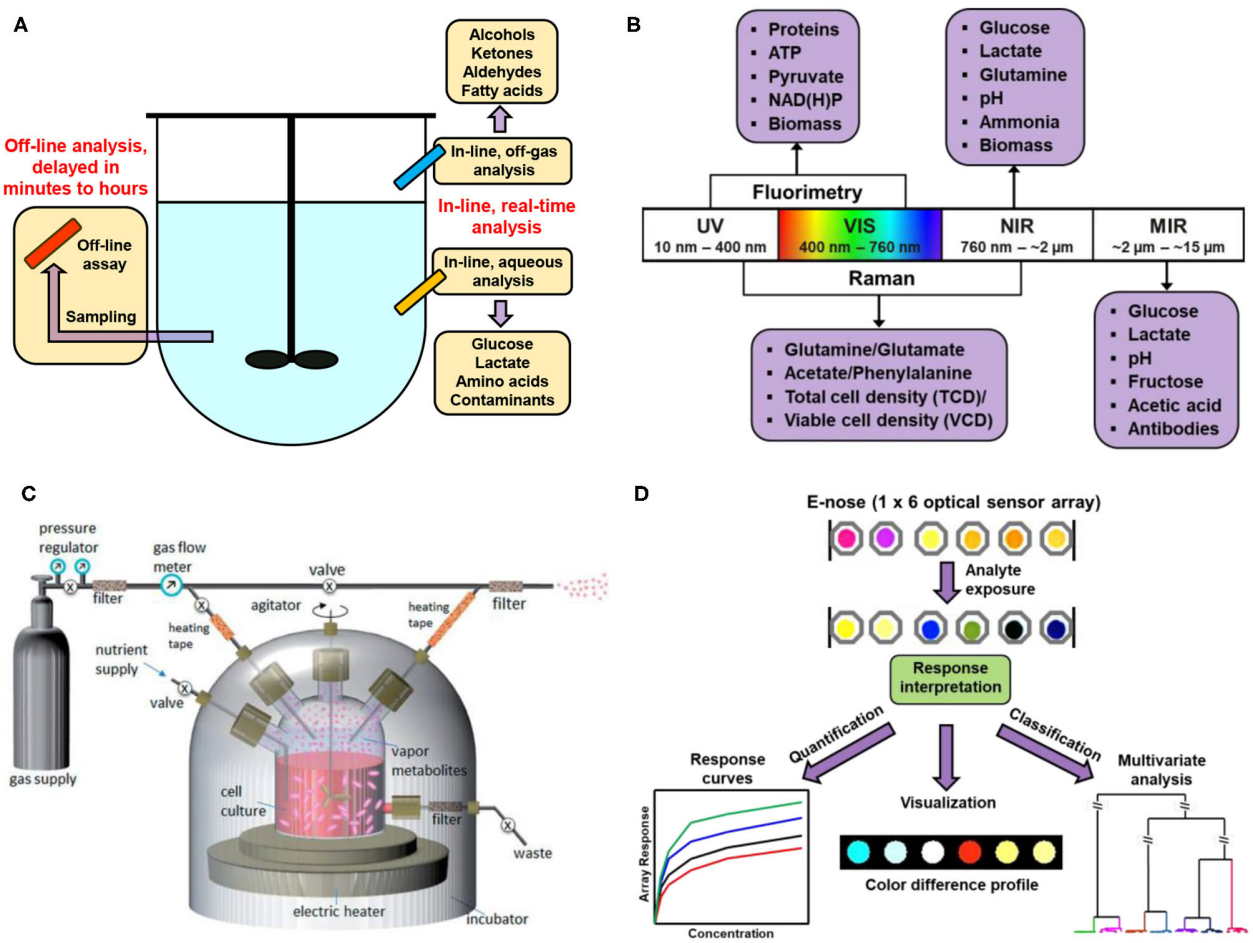
Illustration schemes of the set-ups for biomanufacturing monitoring. **(A)** Scheme of in-line vs. off-line analysis of bioreactors. **(B)** Scheme of the mechanism and interpretation of a typical electronic nose (E-nose) system made of optically responsive sensors. **(C)** Headspace VOC analysis of a growing cell culture in a bioreactor using ambient mass spectrometry. Reproduced with permission from Chingin et al. (2014). Copyright Royal Society of Chemistry 2014. **(D)** Illustration of electronic noses and pattern recognition.

Methods for the measurement of bioprocess variables can be generally divided into two broad groups: on-line and off-line measurements (Figure 1A) (Lourenço et al., 2012; Zhao et al., 2015). On-line measurements require the instrument to be present in the line of bioprocess stream to ensure real-time analysis with instant data output, while off-line measurements deal with sample separation from a bioreactor for measurement at a discrete location or time point. Instrumentation selected for on-line or off-line measurements may be invasive that requires the penetration of probes into the fluid medium (e.g., to read pH and dissolved oxygen), or non-invasive that allows the measurement to be performed without breaking the boundary between the bioprocess stream and the surrounded environment (e.g., headspace gas analysis and aqueous-phase spectroscopic methods).

To date, standard methods for monitoring the status of typical biomanufacturing processes involve the biomolecular quantification using PCR or ELISA techniques (Riahi et al., 2016; Valente et al., 2018; de Bournonville et al., 2019), which is sensitive and specific but dependent on cumbersome analyzing protocols. Other methods based on measuring the content of dissolved oxygen, carbon dioxide or pH have proven ineffective as they usually predict the occurrence of abnormal states (e.g., contamination) at very late stage of cell cultivation (Bachinger et al., 2002). Moreover, those methods sometimes are not reliable as it could be difficult to observe subtle differences in case an in-line bioreactor control system is used to adjust deviations in oxygen or pH. Other indirect analyses of relevant aqueous or gaseous analytes in a production container, however, involve the complicated and cumbersome incubation of a media sample drawn from the bioreactor. Consequently, the success in early diagnosis of biomanufacturing status is largely dependent on the development of new analytical devices that can perform real-time detection of key metabolites in any cell cultivation process.

Off-gas analysis is a simple, rapid and non-invasive approach for monitoring cell status during bioproduction. Although such gas detection focuses mainly on oxygen and carbon dioxide, other characteristic volatile compounds are also of great interest to analytical chemists or biologists. It is well-known that the cultivation of a particular cell culture produces a diverse set of volatile organic chemicals (VOCs) as featured metabolites. A wide variety of standard analytical techniques have been applied to the determination of the identities and contents of different gaseous targets, including gas chromatography (GC) (Langejuergen et al., 2015; Lavra et al., 2015; Tang et al., 2017), mass spectrometry (MS) (Schmidberger and Huber, 2013; Chingin et al., 2014; Chippendale et al., 2014; Schmidberger et al., 2014), near-infrared (NIR) spectroscopy (Cimander and Mandenius, 2002; Zhang et al., 2009; Nespeca et al., 2017; Zavala-Ortiz et al., 2020), and multi-wavelength fluorimetry (Faassen and Hitzmann, 2015; Rowland-Jones et al., 2017). Most of those methods, however, suffer from one or more of the drawbacks below: demand on bulk instrumentation, lack of portability, high cost, and long-term sample preparation and analysis. A facile and cost-effective profiling of biological media for precise identification of key components and correlation of the fingerprint to physiological parameters measured by gold standard analytical techniques is highly desirable.

Developing detection methods for continuous and real-time analysis of effluent off-gases has consequently become a pressing demand. The array-based sensors, or “electronic noses” (Hines et al., 1999; Rock et al., 2008; Berna et al., 2009), aim to construct a class of sensor elements with diverse physical and chemical properties for molecular recognition. The first example of an artificial nose was reported by Persaud et al. that mimicked the biological olfactory system using three metal oxides as semiconductor transducers to detect similar biological mixtures (Persaud and Dodd, 1982). This was one of the earliest attempts to use sensor arrays and to successfully distinguish an extensive range of odors. Since then, a similar idea pertaining to array-based sensing technology using an increasing number of analytical devices with cross-reactive sensing elements have emerged that show impressive potentials in sensing applications ranging from environmental monitoring (Su et al., 2012; Wei et al., 2014; Jayawardane et al., 2015), to security screening (Hu et al., 2014; Bright et al., 2015; Li et al., 2015), to biomedical diagnosis (Konvalina and Haick, 2014; Ulrike and Hossam, 2014; Huang et al., 2015; Tomita et al., 2016), and to food inspection (Maynor et al., 2007; Narsaiah et al., 2012). Innovations in relevant areas are continuing and improvements in sensing technology are constantly needed for resolving limitations of traditional electronic noses that heavily rely on non-specific physical adsorptions of analyte molecules, such as to enhance sensitivity (i.e., limit of detection, or LOD) or selectivity (i.e., limit of recognition, or LOR) of relevant VOCs (Feuz et al., 2010; Li et al., 2019). Array-based optical sensing (i.e., optoelectronic noses) that integrates a diverse range of chemoresponsive and photoactive sensing elements with portable imaging tools proves to be an excellent technique for those goals.

In an attempt to demonstrate the advances and challenges met in the field of bioprocess assessment, we will extensively review the recent development of state-of-the-art techniques for analysis of key components in biomanufacturing process, and mainly focus on optical methods, mass spectrometry, and modern electronic noses. We will cover significant progresses in on-line or off-line monitoring devices and their applications in quality control of typical examples of microbial and mammalian cell cultivation processes, particularly by analyzing the characteristic volatile compounds. Finally, a systematic discussion will be carried out regarding the limitations and future perspectives in bioengineering monitoring technologies.

## Major Targets in Biomanufacturing Monitoring

### Aqueous Analytes

Biomolecules such as glucose, lactate, and amino acid (Schmidt et al., 1998; Roychoudhury et al., 2007; Fan et al., 2016) are important nutrients or metabolites in biomanufacturing. Environmental parameters such as pH, dissolved O_2_ or CO_2_ also play pivotal roles on the growth of cell culture media (Meunier et al., 2016; Michl et al., 2019). Monitoring of those major targets during cell culture cultivation is of great value for the regulation and management of biological production. The complexity of the culture medium, however, poses a great challenge to the real-time detection and analysis of target molecules. Especially for off-line methods, pretreatment of culture medium such as filtration and isolation could significantly improve the detection sensitivity and specificity. Nevertheless, sampling frequency (once every 12–24 h) in off-line contexts is often too low to monitor highly dynamic metabolic processes (Tulsyan et al., 2019), which may result in limited spatiotemporal resolution of the cell metabolic features. Moreover, the pretreatment procedures are usually time-consuming and laborious, and the cultivation fluid is subject to contamination caused by repetitive sampling.

The on-line monitoring methods are expected to resolve those problems mentioned above. Since the detecting units are integrated in the reactor, on-line monitoring methods require no additional steps for sample separation and purification, thus significantly simplifying the analytical procedures and reducing the possibility of sample contamination. In this respect, the non-invasive and contactless spectroscopic techniques are more widely employed in industrial applications. For example, the ultrasound-assisted near-infrared spectroscopy as developed by Kambayashi et al. (2020) used ultrasonic standing waves to generate acoustic radiation force and concentrate the suspended targets at the nodal planes, so that the incoming near-infrared light could precisely quantify target molecules in the colloidal suspension. The measurement error of glucose using this method was as low as 0.6%. In addition, owing to fast data acquisition, high accuracy, and capability of multiparameter analysis, Raman spectroscopy is considered a promising tool in monitoring a wide range of aqueous biological samples (Shaw et al., 1999). For instance, Santos et al. (2018) monitored monoclonal antibody (mAb) cultivations *in situ* using Raman spectroscopy by adjusting calibration models; an automatic and real-time calibration framework was established by Tulsyan et al. (2019) that enabled the integration of traditional Raman models in variable culturing conditions without specific calibration steps, thus increasing the feasibility of Raman techniques in inspecting culture media with highly changeable composition.

Combining with machine-learning techniques (Li et al., 2020), Raman spectroscopy allows for the detection of a large number of analytes or metabolic indices simultaneously, including glucose, glutamate, lactate, metal ions and anions, cell viability and density, which permits multidimensional data analysis for comprehensive profiling of the biological signatures (Henriques et al., 2009). Other optical measurements in bioengineering involve UV/Vis (Noui et al., 2002), mid-infrared or fluorescence spectroscopy (Figure 1B); electrical approaches include terahertz spectroscopy (Plusquellic et al., 2007) and electrochemical sensors (Joo and Brown, 2008). Each technique has its own advantages and limitations that call for further optimization in detection mechanism and instrumentation.

### Volatile Organic Chemicals

Carbon dioxide and oxygen are most commonly targeted volatiles in the off-gas stream, and studies of CO_2_ or O_2_ monitoring using infrared (Cimander and Mandenius, 2002) or paramagnetic (Parente et al., 2004) spectrometry have been well-established. In addition to regular gas emissions from a general bioprocess, there are a wide variety of VOCs with distinctive chemical functionalities for targeting. For example, ethanol, methanol, and isoprenoids are abundantly present in various microbial production processes, and are among the most commonly measured metabolic species in bioproduction (Wang et al., 2020).

Unlike microbial species, mammalian cells can feed on lipids and produce accordingly different classes of metabolites that are rarely found in the culture of microorganisms (Fernández-García et al., 2020). Those products typically consists of ketones, long-chain (> C5) alcohols, alkanes or alkenes, esters, etc. The unique composition of emitted VOCs can be utilized for discrimination of different mammalian cell lines, and examined by an extensive selection of analytical methods, including gas chromatography (Filipiak et al., 2010), optical spectroscopy, mass spectroscopy (MS) (Biasioli et al., 2011), and electronic noses (E-noses) comprising an array of metal oxide or conductive polymer sensors. Recently, proton transfer reaction - mass spectroscopy (PTR-MS) (Brunner et al., 2010) has shown great potential in the detection of VOCs during fed-batch cultivation of recombinant CHO cell cultures (Schmidberger et al., 2014). By applying a soft ionization technology, this technique can avoid producing excessive molecular fragments and therefore simplify spectral interpretation. The method is able to identify eight most abundant VOCs in the bioreactor off-gases, including small molecules such as methanol, acetaldehyde, as well as some long-chain organic species such as 3- or 4-methyl-2-pentanone, hexanoic acid, or C6 ester. The study demonstrates the great advantage of PTR-MS technology in off-gas monitoring of cultivated cell cultures over electronic noses in terms of chemical specificity, and over gas chromatographic methods in terms of data accuracy and discriminatory efficiency.

## Analytical Methods

Modern techniques in bioengineering are urgently needed for a total bioprocess assessment, aiming significant transitions from batch to on-line and instantaneous monitoring (Teixeira et al., 2009; Roch and Mandenius, 2016; Holzberg et al., 2018). Conventional techniques constantly used for bioprocess monitoring are well-established yet relatively outdated (e.g., the classical Clark electrodes), thus demanding the invention of novel and easy-to-use devices. In this context, the utilization of analytical methods is expanding rapidly from laborious PCR/ELISA methods or chromatographic/spectroscopic systems to recently developed electronic noses/tongues using electrochemical, optical or other solid-state sensors. In this section, we will summarize several most common approaches for biomanufacturing analysis.

### Gas Chromatography (GC)

GC is one of the most popular and active analytical technologies in bioengineering (Mcnair and Miller, 2010). The analytes of interest could be in gas, liquid or solid, with molecular weight ranging from 2 to 1,000 Da, which has shown extensive applications in food inspection (Bianchi et al., 2006), pesticide detection (Zhang et al., 2006), environmental monitoring (Viola et al., 2019), forensic investigation (Alexandrino et al., 2019), and quality control of petroleum products (Coutinho et al., 2018). In addition, GC analysis is widely used in the inspection of human organ functions (Young et al., 2019) and screening of cancer cells, such as hematological malignancies (Tang et al., 2017) and breast cancer (Lavra et al., 2015). GC is also considered the gold standard technique for biomanufacturing monitoring. For example, McConnell and Antoniewicz (2016) exploited GC with MS detector (GC-MS) to measure the carbohydrate composition in the culture medium of *chlorella vulgaris*. Compared to former methods for carbohydrate analysis, GC is advantageous in data reproducibility, accuracy, and the capability of multiplexed detection. Recently developed two-dimensional GC-MS benefits from two-step separation (apolar vs. polar) that greatly improves the spatiotemporal resolution of GC (Yu et al., 2017). However, particularly for off-line analysis, sample collection and pretreatment would complicate the analyzing procedures, and even bring in unexpected contamination. As an indirect but effective method, headspace sampling is a field-deployable method that can integrate the GC system directly into the bioreactor, so as to skip the sampling step, speed up analyzing rate, and reduce the possibility of sample contamination. The concept of monitoring characteristic VOCs have been successfully demonstrated in the identification of microalgae (Guidetti Vendruscolo et al., 2019) and pathogenic bacteria (Chen et al., 2017). With the continuous progress on new columns (packed or capillary), separation mechanisms, and expansion of molecular data library, it is expected to further improve GC detection sensitivity and resolution of relevant species regarding particular applications in bioengineering, such as proteomics/lipidomics.

### Mass Spectrometry (MS)

The rapid development of MS since the past few decades have dramatically expanded the fields research to many aspects in bioengineering, such as cellular proteomics or metabolomics (Ali et al., 2018; MacMullan et al., 2019; Li and Shui, 2020); in particular, the invention of matrix assisted laser desorption ionization (MALDI)-MS (Hillenkamp et al., 1991) pave the path for rapid and simple fingerprinting of biomacromolecules (Kawano et al., 2019). Advances in recent years have led to a variety of novel ionization methods or detection mechanisms for biomanufacturing assessment. As a typical example, GC-MS tandem technique is considered the gold standard for general VOC analyses (Romano et al., 2015). The recently developed direct injection (i.e., ambient) mass spectrometry (DI-MS) requires no complicated steps for sample separation or purification; gaseous analytes from the headspace could be introduced directly into the mass spectrometry system, thus significantly facilitating the sample analysis procedures (Figure 1C). Among various DI-MS techniques, proton transfer reaction-MS (PTR-MS) (Hansel et al., 1995) and selected ion flow tube-MS (SIFT-MS) (La Nasa et al., 2019) represent the cutting-edge technologies, which takes advantages of relatively simple instrumentation and controllable ionization conditions. Combining with time-of-flight (TOF) mass analyzer, the detection sensitivity could reach parts per trillion by volume (pptv) level, with m/Δm up to 15,000 (Soukoulis et al., 2010). Examples of MS techniques for bioproduction analysis were listed in Table 1.

**Table 1:** List of techniques of mass spectrometry or electronic noses employed for off-gas monitoring of specific cell cultivation.

**Cell culture**	**Bioprocess monitored**	**Mass spectrometry**	**Volatiles detected**	**References**
**MASS SPECTROMETRY**				
*Escherichia coli* JM 109	Accidental infection by *comamonas testosteroni*	Time-resolved SIFT-MS	Ethanol, acetaldehyde, hydrogen sulfide and ammonia	Chippendale et al., 2011
JEKO and SHI-1 cell lines	Tissue culture	SPME-GC–MS	Dimethyl sulfide, 2,4-dimethylheptane, methylbenzene, *o*-xylene, dodecane, 1,3-di-tert-butylbenzene, ethanol, hexanal, and benzaldehyde	Tang et al., 2017
*Streptococcus thermophilus*, and *bulgaricus*	Lactic acid fermentation of milk	PTR-TOF-MS	Acetaldehyde, 2-propanone, diacetyl, acetoin, etc.	Soukoulis et al., 2010
A549 epithelial cell line, and *Pseudomonas aeruginosa*	Co-culture of lung epithelial cell line with *Pseudomonas aeruginosa*	thermal desorption-GC-MS	3-methyl-1-butanol, acetone, ethylidene cyclopropane, ethanol, tert-butyl ethyl ether, methyl tert butyl ether, etc.	Lawal et al., 2018
*Lactobacillus fermentum* IMDO130101	Submerged fermentations	SIFT-MS	Ethanol	Van Kerrebroeck et al., 2015
*Lactobacillus pentosus*	Milk fermentation	SPME-GC-MS	Twenty-four kinds of VOCs including acetaldehyde, acetone, etc.	Pan et al., 2014
*Nodulisporium* TI-13	Beet pulp fermentation	PTR-MS	Ethanol, methanol, acetaldehyde, terpenes, and terpenoids	Schoen et al., 2016
*Escherichia coli* HMS174(DE3)	*Escherichia coli* cultivation	PTR-MS	Total VOCs	Luchner et al., 2012
*S. cerevisiae*	Mead fermentation	PTR-MS	Total VOCs	Cuenca et al., 2016
Eleven lactic acid bacteria including *E. casselliflavus* FMAC163, et al.	*Caciocavallo Palermitano* cheese fermentation	SPME-GC-MS	Alcohol, aldehydes, ketones, esters, aromatic, organic acid, hydrocarbons, etc.	Guarrasi et al., 2017
**Cell culture**	**Bioprocess monitored**	**E-nose type**	**Volatiles detected**	**References**
**E-NOSE**				
Recombinant CHO	Early detection of bacterial infection	10 MOSFET, 12 MOS	O_2_	Bachinger et al., 2002
*Saccharomyces cerevisiae* (a yeast)	Non-volatile metabolites during fermentation	18 MOS	Ethanol, acetic acid, and acetaldehyde	Liden et al., 2000; Calderon-Santoyo et al., 2010
Recombinant *Escherichia coli*	Metabolic burden	19 MOSFET, 10 MOS	Biomass and other non-specific gases	Bachinger et al., 2001
CHO and Sf-9 insect cell culture	Microbial and viral contaminants	10 MOSFET, 6 MOS	Acetic acid, ammonia, acetone, and ethanol	Kreij et al., 2005
*Morinda citrifolia* and *Nicotiana tabacum* (plant cells)	Biomass and metabolite concentration	19 MOS and a CO_2_ sensor	Biomass and other non-specific gases	Komaraiah et al., 2004
*Bacillus subtilis*	Sporulation events	10 MOSFET, 6 MOS	Volatiles from spores	Clemente et al., 2008
*Lactobacillus fermentum* Ogi E1	Lactic fermentation	18 MOS	Ethanol	Calderon-Santoyo et al., 2013
Recombinant CHO	Cellular state transitions	10 MOSFET, 6 MOS	Biomass and other non-specific gases	Bachinger et al., 2000
*Acidithiobacillus thiooxidans*	Maturation of air-lift bioreactors	11 MOS	Alcohols, hydrocarbons, sulfur compounds, etc.	Rosi et al., 2012

*SIFT, selected ion flow tube; PTR, proton transfer reaction; SPME, solid-phase microextraction; MOSFET, metal-oxide-semiconductor field-effect transistor; MOS, metal oxide semiconductor*.

### Electronic Nose

The advent of electronic nose (E-nose) has provided alternative tools for sensing VOCs in the bioreactors. E-noses employed for this purpose generally consist of an array of responsive gas sensors, each of which allows for the determination of both the identity and concentration of unknown gas analytes. The unique pattern representing the overall sensor response caused by the exposure of VOCs can be used as a “fingerprint” for the identification of cell cultures of microorganisms or mammalian cell lines (Figure 1D). Pattern recognition based on a dozen of sensor elements, in a sense, is more accurate than the identification made by only one or two sensors. The distinctions among different cell cultures can be demonstrated by a diverse range of clustering and classification methods. Clustering methods seek to describe a dataset into groups, or clusters; classification methods attempt to predict information about an unknown sample based on previously acquired data. Common statistical methods for biomanufacturing monitoring include: principal component analysis (PCA) (Albrecht et al., 2018; Chen et al., 2020) linear discriminant analysis (LDA) (Wang et al., 2012; Silva et al., 2017), partial least square (PLS) regression/discrimination analysis (Kammies et al., 2016; Matthews et al., 2016; McCartney et al., 2019; Pontius et al., 2020; Zavala-Ortiz et al., 2020), and artificial neural network (ANN) (López et al., 2017; Oyetunde et al., 2018).

Most e-noses for volatile gas measurement generally rely on the adsorption of gas molecules to the surface of sensors. The sensing instrumentation usually consists of an array of sensors that have different binding capabilities to gaseous analytes and output electrical signals (e.g., changes in current, voltage or resistance) as a result of the selective adsorption. Common E-noses include conductive polymers (Kiilerich-Pedersen et al., 2011), metal oxide semiconductors (MOS) (Liden et al., 2000; Calderon-Santoyo et al., 2010), metal oxide semiconductor field effect transistors (MOSFET) (Bachinger et al., 2001; Komaraiah et al., 2004; Kreij et al., 2005; Clemente et al., 2008), quartz crystal microbalances (QCM) (Shen et al., 2007), surface acoustic wave (SAW) devices (Rocha-Gaso et al., 2009), and chemiresistive or amperometric sensors (Chiu and Tang, 2013; Ahmed et al., 2014). Compared to single sensing, the highly integrated, multiplexing sensor array satisfies the requirements for both identification and quantification of targeted VOCs.

Due to the ease of sensor fabrication and deployment, E-noses have found extensive applications in the analysis of off-gas emissions from the bioproduction of a large number of microorganisms, mammalian or insect cell lines (Namdev et al., 1998; Bachinger and Mandenius, 2000; Mandenius, 2000; Cimander et al., 2002). An early study was report by Liden et al. (2000) to quantify major metabolites during fermentation of a yeast, *Saccharomyces cerevisiae*, through on-line analysis of the off-gas emission using a set of 10 MOSFETs (e.g., ethanol, acetaldehyde, etc.). Other successful applications of E-noses in detecting mammalian cell lines have been continuously reported (Calderon-Santoyo et al., 2010; Cuypers and Lieberzeit, 2018). Examples involve the monitoring of metabolic burden in the fermentation process of a recombinant *Escherichia coli* (Bachinger et al., 2001), the early detection of bacterial/fungal contaminations in mammalian (e.g., recombinant CHO cells) and in insect (e.g., recombinant Sf-9 cell for protein production) cell lines (Bachinger et al., 2002; Kreij et al., 2005), as well as the quantification of plant cell cultures (e.g., *Morinda citrifolia* and *Nicotiana tabacum*) (Komaraiah et al., 2004), and of spore concentration in the cultures of *Bacillus subtilis*, a species for oral bacteriotherapy of gastrointestinal diseases (Clemente et al., 2008). Different E-noses employed for microbial and mammalian cell detection with various purposes were listed in Table 1.

### Optoelectronic Nose

The thriving development of novel techniques in chemical sensing (Albert et al., 2000; Nakamoto and Ishida, 2008; Wu et al., 2015) has led to the availability of a more integrative type of sensory tools as alternatives to traditional electronic noses, namely the “optoelectronic nose” (OE-nose) (Rakow and Suslick, 2000). OE-nose is a class of optical sensor arrays based on chemoresponsive colorants to probe chromogenic or fluorimetric changes induced by specific interactions. This provides a high dimensionality to chemical sensing that permits high sensitivity (often down to ppb or even ppt levels), impressive discrimination among very similar odorants and superb fingerprinting of extremely similar mixtures over a wide range of categories, both in gaseous and liquid phases. Optical sensor arrays therefore sufficiently overcome the limitations of traditional array based sensors that solely depend on physical adsorption or non-specific chemical interactions.

Optical array sensing has shown excellent performance in the detection and identification of diverse analytes, ranging from chemical hazards (Li and Suslick, 2019) to medical biomarkers (Wang et al., 2016; Li and Suslick, 2018), and to food additives (Schaude et al., 2017). Likewise, this method can be promisingly applied to the analysis of effluent VOCs from the cultures accumulated in the headspace of bioreactors, as a way of monitoring microbial or cellular activities. Suslick et al. have designed colorimetric sensor arrays coupled to optoelectronic readers for the identification of different bacteria and fungi based on the off-gas analysis (Carey et al., 2011; Li et al., 2019). The concept of such detector is to incorporate chemoresponsive colorants in an array of sensors, which undergoes significant colorimetric or fluorimetric changes in response to VOCs through an extensive range of chemical interactions including bond formation or ligand coordination, proton acid-base interactions, hydrogen or halogen bonding, charge-transfer and π-π stacking, etc. The array is digitally imaged before and during exposure, and an optical difference profile is generated in real time by subtracting the before-exposure image from images after gas exposure. Simple pattern recognition techniques give essentially error-free recognition of gas analytes based on a numerical library (3N vectors of red-green-blue (RGB) difference changes). Such libraries require very little memory and are easily updatable and transferrable, thus enabling the instant and continuous monitoring of biological production.

## Discussion

Biomanufacturing industry has received remarkable improvements in process efficiency and productivity over the past decade. Efforts for bioprocess inspection are placed on enhancing process robustness and product quality using a wide range of *in-situ* analytical techniques. For effective bioprocess monitoring, it is critical to characterize physiological states and measure main process variables, such as key nutrients and metabolites in the aqueous phase, or off-gas effluents as characteristic VOCs. Numerous chromatographic and spectroscopic techniques, particularly gas chromatography and mass spectrometry, have shown impressive potentials in real-time profiling of the cell culture status. Meanwhile, the optimization of chemometric models that provide quantitative and predictable correlation between the spectra pattern and targeted key metabolites is also of great importance. Much of the endeavors have been focused on classical analytical instrumentation and off-line measurements, while little has been related to *in-situ* monitoring protocols.

Admitted, a complete quantitative component-by-component analysis using an effective analytical method is always desirable. Nevertheless, one seldom really wants to know clearly the identities of hundreds of compounds present in a complex mixture, such as the chemical composition of a biomanufacturing media. Alternatively, those goals are better fulfilled by a highly discriminant fingerprinting of the entire mixture. Such fingerprinting strategies permit comparison to standards, identification of chemical class or origin of the species, and correlation of the fingerprint to properties determined by other standard techniques. In the case where component-by-component analysis is required, the tandem approach that combine a separation technique (e.g., various chromatographic approaches or electrophoresis) with an analysis technique is typically employed. Sensor array techniques such as electronic or optoelectronic noses are most commonly used for direct fingerprinting of complex mixtures without sophisticated instrumental operation and complicated sample separation.

Due to miniaturized and multiplexed features, E-noses have received tremendous attention in recent years in connection with a broad range of bioengineering fields. E-nose devices are constructed from an array of engineered sensors that provide a pattern of electronic signals in response to a given analyte; therefore, they are distinctive from other techniques for biochemical analysis in that they are primarily designed to recognize gas mixtures as a whole without the need for identifying individual chemical species in the mixture. For this reason, E-noses are not intended for determining the chemical composition of a complex gas mixture, but rather are most useful for identifying the produced “chemical bouquet.” Even for highly close gas mixtures from very similar species, it is possible to tell their subtle differences using effective E-noses.

Despite the great success achieved by E-noses in monitoring cellular physiology, traditional E-noses often suffer from low chemical specificity and sensitivity due to simple or non-specific interactions with the biomarkers (i.e., physical adsorption). The other shortcoming lies in the sensor drift, which leads to poor stability and reproducibility in sensor outputs. Moreover, E-noses could be easily interfered by ambient humidity or pressure changes, which demand repetitive calibration of the sensor devices. In addition, some E-noses have to be operated under restricted conditions. For example, metal oxide semiconductors are generally required to work at high temperatures (generally over 100°C) with a large amount of power consumption. Therefore, there remains an urgent demand for improving sensing performance of the current electronic sensors.

Array-based optical sensing, on the other hand, attempts to probe mostly the chemical reactivity of analytes rather than their physical properties. That provides a high dimensionality for biosensing purposes and enables high sensitivity (often down to ppb levels) and remarkable discrimination among highly similar targets in gaseous phases. The optoelectronic noses made of chromogenic or fluorometric elements therefore overcome the limitations of traditional electronic sensors that solely depend on physisorption or non-specific chemical interactions. Such concept of optical array sensing has shown excellent performance in the detection and identification of a diverse set of analytes, ranging from industrial toxins to energetic explosives, to human biomarkers, and of course, to products of great relevance to biomanufacturing processes.

In conclusion, instead of the conventional and stepwise off-line sample analyses that have to be conducted in laboratory settings, developing non-destructive optical sensing techniques for real-time monitoring of effluent gases in large-scale bioreactors is highly desirable. Although there is very limited number of research reported on the use of optical array sensing for profiling featured headspace volatiles from a typical bioproduction process, we expect that an increasing number of optical sensor array are likely to be developed for this particular application, and more advanced strategies along with a significantly thriving market in this area are likely to be established in the near future.

## Author Contributions

XC and ZL took the lead in writing the manuscript. All authors provided critical feedback, helped shape the manuscript, and references.

## Conflict of Interest

The authors declare that the research was conducted in the absence of any commercial or financial relationships that could be construed as a potential conflict of interest.
